# Coastal wetlands can be saved from sea level rise by recreating past tidal regimes

**DOI:** 10.1038/s41598-021-80977-3

**Published:** 2021-01-13

**Authors:** Mahmood Sadat-Noori, Caleb Rankin, Duncan Rayner, Valentin Heimhuber, Troy Gaston, Christopher Drummond, Anita Chalmers, Danial Khojasteh, William Glamore

**Affiliations:** 1 Water Research Laboratory, School of Civil & Environmental Engineering, UNSW Sydney, 110 King St., Manly Vale, Sydney, NSW 2093 Australia; 2 School of Environmental and Life Sciences, University of Newcastle, Newcastle, NSW Australia

**Keywords:** Environmental impact, Climate-change mitigation

## Abstract

Climate change driven Sea Level Rise (SLR) is creating a major global environmental crisis in coastal ecosystems, however, limited practical solutions are provided to prevent or mitigate the impacts. Here, we propose a novel eco-engineering solution to protect highly valued vegetated intertidal ecosystems. The new ‘Tidal Replicate Method’ involves the creation of a synthetic tidal regime that mimics the desired hydroperiod for intertidal wetlands. This synthetic tidal regime can then be applied via automated tidal control systems, “SmartGates”, at suitable locations. As a proof of concept study, this method was applied at an intertidal wetland with the aim of restabilising saltmarsh vegetation at a location representative of SLR. Results from aerial drone surveys and on-ground vegetation sampling indicated that the Tidal Replicate Method effectively established saltmarsh onsite over a 3-year period of post-restoration, showing the method is able to protect endangered intertidal ecosystems from submersion. If applied globally, this method can protect high value coastal wetlands with similar environmental settings, including over 1,184,000 ha of Ramsar coastal wetlands. This equates to a saving of US$230 billion in ecosystem services per year. This solution can play an important role in the global effort to conserve coastal wetlands under accelerating SLR.

## Introduction

Vegetated intertidal ecosystems, such as mangroves and saltmarshes, are located at the interface between land and sea. These ecosystems are vital to the ecological functioning of estuaries and provide enormous ecosystem services^1^, including the provision of habitat^2^, supporting commercial and non-commercial fisheries^3^, providing water storage and purification^4^, flood regulation^5^ and carbon sequestration^6,7^. These services either directly or indirectly influence human well-being, highlighting that vegetated intertidal ecosystems are significantly valuable and economically important^8–10^. At the same time, these ecosystems are among the most vulnerable environments to sea level rise (SLR) as they are located adjacent to the open sea, have a low-lying landscape and dense vegetation population^11^. Significant losses in intertidal ecosystems have been reported over the last decades due to human activities^12,13^. For example, during the period 1984–2016, approximately 16% of the global surface area covered by intertidal flats was lost, primarily due to human activities and regionally-variable SLR^14–16^.

Recent updated IPCC projections of global mean SLR by 2100 range from 0.61 to 1.10 m (RCP 8.5, likely range^17,18^) and a number of studies suggest that, due to large uncertainties in the stability of Greenland and Antarctic ice sheets, scenarios of over 2 m by 2100 are within the possible range^18–20^. The already accelerating rates of SLR^21^ pose a growing threat to intertidal wetlands and studies predict the submergence of 20–78% of worldwide coastal wetlands by 2100^22^. On the contrary, a number of recent studies suggest that increases in the global intertidal wetland area is possible under SLR^6,10,23^, however, these potential increases rely on accretion rates (vertical) and the availability of space to accommodate the landward (horizontal) migration of wetlands.

In many coastal settings, the horizontal migration of wetlands towards more elevated surrounding areas is not possible due to physical barriers, environmental conditions, or socio-economic complexities (e.g. private land ownership). Additionally, vertical accretion rates may be limited by sediment supply or the organic matter accumulation rate. While there is ongoing uncertainty regarding these processes, the widespread loss of valuable, healthy, vegetated intertidal ecosystems due to SLR (including many Ramsar listed wetlands of international importance) is a likely outcome in many locations^24^. As such, it is essential to develop a sustainable solution to preserve high value vegetated intertidal ecosystems from SLR impacts.

Current literature suggests that a major global environmental crisis in coastal ecosystems is underway, due to the loss of intertidal ecosystems, but offers limited practical solutions to prevent or mitigate the impacts^12,14,25^. The four most common options for managing the impacts of SLR on intertidal ecosystems are^26–29^:

1. status quo (maintaining existing management strategy);
2. retreat landwards (horizontal migration);
3. sediment supply (vertical accretion) and
4. protection/defence measures.

Option 1 (status quo) also considers the ‘no action’ strategy, which may lead to the ecosystem perishing (depending on accretion rates) as it becomes permanently inundated. Option 2 (horizontal migration) has significant uncertainty regarding the availability of space, sediment type, slope and plant physiological response^6,30^. This ‘retreat’ option is particularly challenging for ecosystems of international importance, such as Ramsar wetlands, where they are geographically fixed in a location and may be limited by the area’s topography or upland barriers. Option 3 (vertical accretion) is also associated with large uncertainties as accretion processes are highly complex and variable across space and time, including inter- and intra-annual variations^31^. Past accretion rates may not be reliable indicators of potential future rates, as they may represent a period of significantly higher or lower suspended sediment delivery in part due to historic anthropogenic activities^32,33^. As such, future accretion rates are challenging to predict. Overall, the uncertainty in accretion rates, presence of physical barriers and land management complexities, suggest both horizontal migration and vertical accretion management strategies may not be a viable solution for managing high priority intertidal ecosystems under SLR^33^.

In intertidal wetlands, where ecosystems are aligned with tidal inundation patterns, future SLR will alter a site’s hydrology and impact existing vegetation communities^26^. One solution to this pressure, is to preserve the existing tidal hydrology by artificially manipulating the tidal regime. In many locations worldwide this could be achieved by implementing a synthetic tide using hydraulic control gates. While alternative methods that minimize intervention, impact and resources should be preferred, this method can be suitable where existing intertidal ecosystems and their services are at risk and no other alternative is feasible.

**Figure 1:**
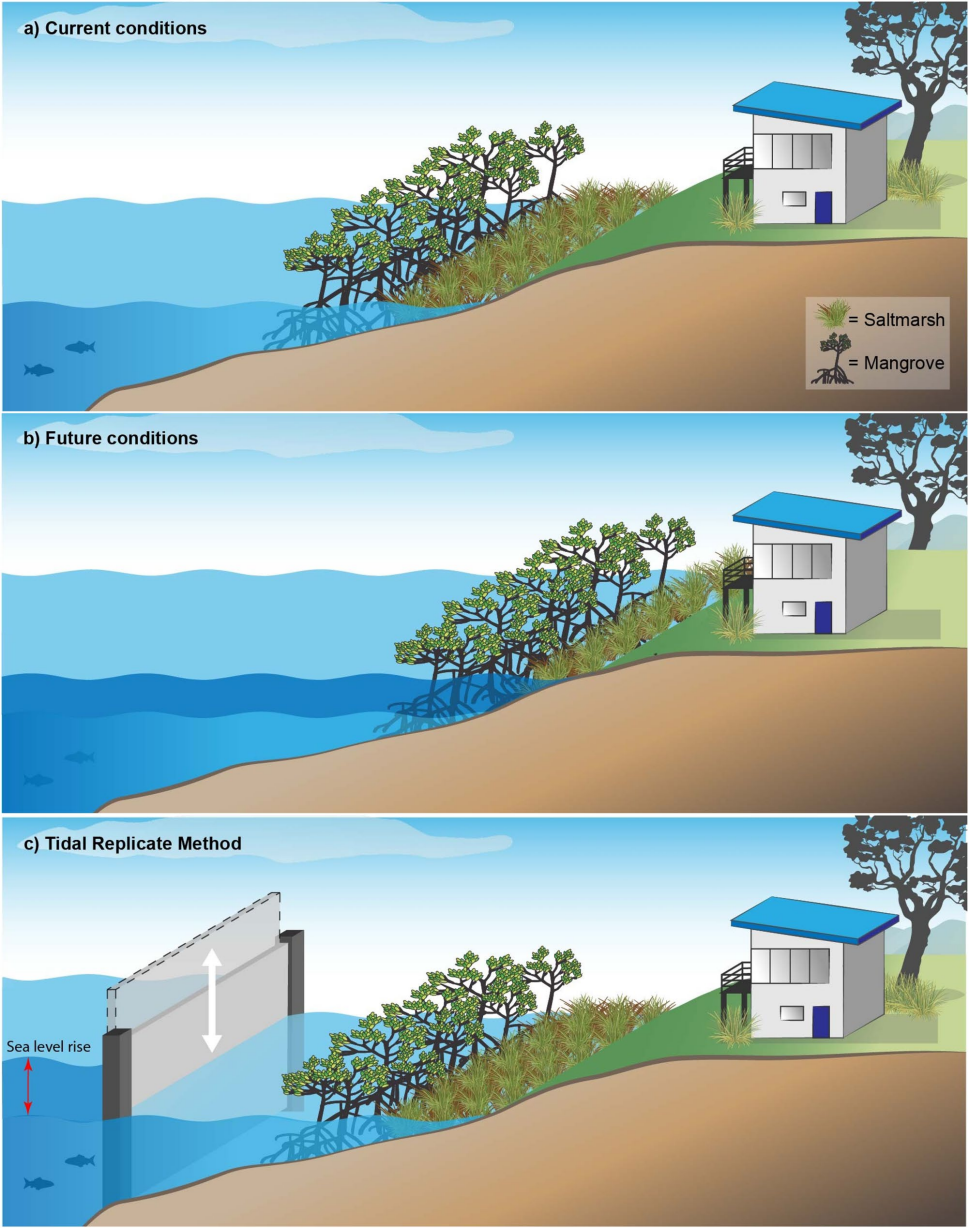
Conceptual diagram showing saltmarsh and mangrove vegetation under (**a**) current conditions, (**b**) future SLR conditions without a solution, and (**c**) future SLR conditions with the Tidal Replicate Method preserving the desired vegetation. The figure was created using Adobe Illustrator 23.0.1 (https://www.adobe.com).

In this study, we present an eco-engineering solution to offset SLR impacts in high priority intertidal ecosystems via a synthetic tidal regime. This is achieved by assessing the existing tidal dynamics of the intertidal ecosystem of interest and then replicating these conditions at a location threatened by elevated sea levels (Supplementary Figure 1). A conceptual diagram illustrating how vegetated intertidal ecosystems can be restored using this “Tidal Replicate Method” is presented in Fig. 1.

The proposed method has the potential to preserve large areas of intertidal wetlands around the world in response to SLR. Focusing on Ramsar wetlands of international importance, we show that this method provides a practical solution to protect many valuable intertidal wetlands from permanent inundation, thereby potentially saving billions of dollars in ecosystem services globally. Additionally, the method has the capacity to work in conjunction with natural accretion rates providing a backup solution if the natural accretion rate is exceeded. Considering the high level of uncertainty related to the potential horizontal migration of intertidal wetlands to more elevated adjacent lands, this eco-engineering solution could play an important role in adaptively managing the global effort to conserve coastal wetlands in the face of accelerating SLR over the twenty-first century.

## Results

### Synthetic tidal regime

The proposed Tidal Replicate Method requires synthesising the tidal dynamics of the desired vegetated intertidal ecosystem, based on hydroperiod of the vegetation species. This involves understanding the hydroperiod conditions of an intertidal community (e.g. saltmarsh or mangroves), including the frequency, depth, and duration of inundation in relation to the elevation of the area of interest. The synthesised tide can then be replicated onsite by installing an automated tidal control system, which we refer to as a ‘SmartGate’, at the entrance of the wetland or connecting channel (Supplementary Figure 2). Through a series of water level triggers, the SmartGate imposes tidal conditions necessary to encourage the recruitment and establishment of target vegetation species.

The synthetic tidal regime is initially developed based on the existing relationship between the intertidal ecosystem and the tidal dynamics. Tidal planes at the site of interest are used and analysed to calculate tidal inundation patterns (for example tidal planes for the Hunter River estuary in eastern Australia are shown in Supplementary Figure 3 and Table 1). The aim of this analysis is to develop a relationship between tidal ranges and vegetation species which, in turn, provides the number of tides per year that would inundate a site and the inundation depth.

The Tidal Replicate Method was applied to a study site over a period of 3 years and results are presented here. At the study site, field survey results showed that saltmarsh is abundant above mean high water (MHW), while mangroves dominate at lower elevations (Supplementary Figure 4). Saltmarsh habitats primarily occurred between MHW and the highest astronomical tide (HAT), with a 50th percentile (median) elevation of + 0.77 m Australian Height Datum (AHD) and a 95th percentile elevation of + 1.1 m AHD. Mangroves occurred throughout the whole tidal envelope, however, the 50th percentile elevation of + 0.44 m AHD was observed from the selected points, with the 95th percentile elevation of + 0.89 m AHD. The ingress of some mangroves into saltmarsh communities was observed at several locations. For the study site, to maximise saltmarsh extent, topographic surveys were used to delineate between the intertidal wetland area and the main tidal channel (crest at + 0.3 m AHD). This elevation trigger ensured that the tide could rise to 0.3 m AHD onsite allowing regular water exchange and connectivity, without impacting the intertidal area. As such, the baseline minimal trigger was set at a threshold of + 0.3 m AHD (Supplementary Figure 5). Thereafter, any water levels above the 0.3 m AHD trigger were set to be indicative of saltmarsh inundation patterns.

Based on the surveys, the median tidal level for saltmarsh at reference sites was + 0.77 m AHD. Therefore, the tidal inundation regime of the reference sites was superimposed onto the study site, with + 0.3 m AHD trigger being the base level. In other words, the tidal regime of the reference sites (with natural saltmarsh vegetation) was replicated at the study site. This resulted in a synthetic tidal regime being created where water levels exceed + 0.3 m AHD 2.8% of the time (equivalent to approximately 110 tides per year exceeding + 0.3 m AHD). This is equivalent to an inundation frequency of water levels above mean spring high tide. To optimise the saltmarsh area and limit mangrove encroachment at the site, additional trigger levels (rather than a single trigger) were created based on the topography of the site and the desired tidal inundation regime (equivalent to the natural tidal regime of reference sites for saltmarsh). Table 1 provides the estimated approximate annual inundation rate for the specified elevations at the study site. These levels were successfully applied onsite via a SmartGate structure over a 3-year period (Supplementary Figure 6). During this time, the surveyed reference sites remained saltmarsh.

**Table 1:** Calculated annual inundation rates for various tidal elevations.

SmartGate trigger water level (m AHD)	Equivalent natural (actual) peak water level^a^	Exceedance probability (%)^b^	Percentage of water levels below peak (%)	Approximate tides per year to reach peak
+ 0.30	0.75	2.8	97.2	600
+ 0.36	0.81	1.6	98.4	50
+ 0.45	0.90	0.3	99.7	45
+ 0.55	1.00	0.01	99.9	18

^a^Based on vegetation elevation.

^b^The duration water level is above the threshold in a natural site.

### Saltmarsh vegetation development/response

**Figure 2:**
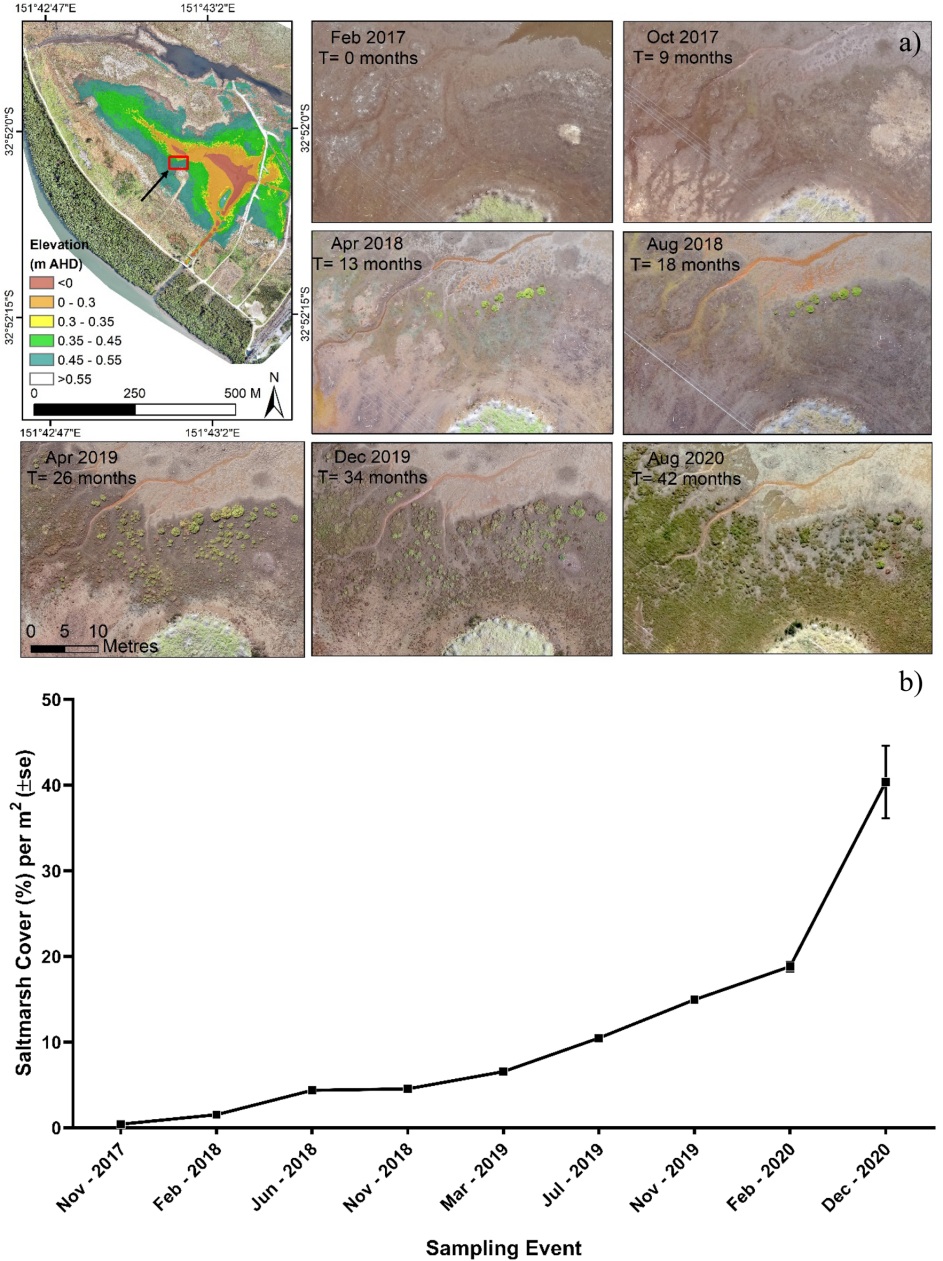
(**a**) Saltmarsh vegetation development over time after implementing the Tidal Replicate Method based on aerial imagery. (Red box in the left-hand side map indicates the area zoomed and illustrated over time.) (**b**) Saltmarsh vegetation surface cover development from the start of the rehabilitation project in September 2017 based on field sampling, highlighting saltmarsh expansion as a result of the Tidal Replicate Method. Map was created using Arc-GIS 10.5 (http://www.esri.com), photos were taken by authors and graph was created using GraphPad 8 (http://www.graphpad.com).

Aerial imagery from drone surveys indicated a positive trend of saltmarsh vegetation extent and distribution since the Tidal Replicate Method was implemented onsite (Fig. 2a). Repeated quadrat vegetation field sampling indicated that the desired saltmarsh species were recruited namely, *Sarcocornia quinqueflora*, *Sporobolus virgincus* and *Suaeda australis*. *Sarcocornia quinqueflora* had the highest recruitment with a 50% increase in cover (m^2^) since the Tidal Replicate Method was implemented (from November 2017 to *December* 2020). Total saltmarsh vegetation cover increased from ~ 0.2% in November 2017, to 45% in December 2020 (Fig. 2b) based on field sampling, indicating the feasibility of the method.

## Discussion

There is limited guidance relevant to the conservation of high value intertidal wetland communities threatened by accelerating SLR^26^. In this study, we applied an eco-engineering solution to a threatened intertidal ecosystem and demonstrated its outcomes 3-years post rehabilitation. As desired, the site, which would have been inundated under natural tidal conditions, has re-established saltmarsh vegetation following the implementation of the Tidal Replicate Method. This indicates that the method is feasible and should be compatible at intertidal wetlands with similar geometry (e.g. one main entrance/exit channel) and shallow water levels.

The concept of controlling the tidal regime through a SmartGate hydraulic structure can be applied to tidal wetlands regardless of their size as long as they meet the geometry and boundary conditions required. For example, this concept was applied at the Ramsar listed Tomago Wetlands site in eastern Australia spanning over 400 ha with similar outcomes of saltmarsh growth and return of migratory shorebirds^34^. Additionally, a range of different physical methods delivering the same concept can be used depending on the value of the ecosystem (i.e. Ramsar wetlands have high value). For example, advanced electrical gates with a larger upfront investment can be used in some locations, whereas low cost buoyant lifting gates can be used to control the hydrology onsite in other locations. In many circumstances, larger upfront costs are required where various risks are identified, and lesser ongoing maintenance is desired.

Retreating landwards and sediment supply are alternative methods that could potentially achieve the same outcomes. However, the method proposed here has several advantages: connectivity with the main tidal channel is preserved and no permanent (fish) barriers are installed (e.g. the system is open to flushing ~ 90% of the time), it preserves onsite soil/sediment characteristics, it can be implemented onsite and modified based on accretion rates, and it typically only requires one piece of infrastructure for its functionality (depending on the site geometry). Further, there is limited ongoing maintenance, it does not require large volumes of exotic foreign sediment to be brought in (which could negatively affect other areas), and it does not impact the existing onsite seedbank (Supplementary Table 2). However, the main benefit of this method is that the synthetic tidal regime can be designed to maintain or create saltmarsh, mangrove, or mudflat ecosystems as well as a specific combination of these, as desired. Additionally, it has the flexibility to adaptively manage the tidal inundation regime over time (e.g. as rehabilitation progresses), with varying land accretion and SLR rates. It is also noteworthy that the ecosystem services (e.g. storm water retention, protection from tidal surge, etc.) provided by saltmarsh vegetation developed using the Tidal Replicate Method should be the same as saltmarsh developed under natural conditions. The main limitation with this method, however, is its limited applicability to intertidal ecosystems located along the open coast or in large oceanic embayments, as a channelised entrance (i.e. hydraulic control point) to the site is required to control the site’s hydrology.

### A comparison with retreating landwards and sediment supply methods

A comparison of the proposed method to the sediment supply and landward migration strategies highlights the value of the Tidal Replicate Method. For instance, the sediment supply method involves landform building via sediment deposition and vertical accretion on areas that are under threat from SLR^35^. The sediment supply method requires the sediment material to be similar with the material naturally found onsite and, hence, may need to be transported from remote locations. In some cases, it requires large quantities of sediment and the process may be prolonged and ongoing^36^. Additionally, sourcing sediment may be challenging and, if dredging is required, significant pumping costs may make this process prohibitive. In contrast, the Tidal Replicate Method overcomes such problems by adjusting the tidal regime to promote the desired conditions onsite for a range of sea level and sediment accretion changes over time.

An alternative option for protecting vegetated intertidal ecosystems is to foster the landwards (or upslope) retreat with SLR. Recent research suggests that in the face of SLR, the provision of upslope accommodation space is more critical for the future global extent of vegetated intertidal ecosystems than vertical accretion^6,37,38^. However, this may not always be a feasible option and depends on firstly, the availability of surrounding low-lying land with suitable elevation, which may be limited by urbanisation, natural geographic boundaries, existing infrastructure and private land ownership^39,40^, and secondly, the political decision-making process regarding the management of these coastal areas (e.g. sediments may not be appropriate for rehabilitation and the timeframes for rehabilitation could be beyond the timing for the wetland retreat).

The landward retreat option is a less desirable approach as it can affect global organic soil carbon accumulation^41^. Existing vegetated intertidal ecosystems may be holding millennia old blue carbon stocks that can be released if such ecosystems are degraded or lost^42^. Additionally, other ecosystems that provide different but specific functions may already exist on the landward side. Landward retreat can place these ecosystems under threat and conservation may need to be considered at some locations. Some sites, like Ramsar listed wetlands, are geographically linked to a location, and cannot be moved as their boundaries are set by law. Many of these sites may have high cultural value and provide services for regional communities^43^ and may need to be preserved.

### Global sea level rise vs accretion rate

Where upland slope retreat is not an option, the ability for any vegetated intertidal ecosystem to adapt to SLR will be largely reliant on the site’s ability to maintain accretion rates in line with SLR. The global mean SLR during the satellite altimetry period (1993–2014) has increased at a rate of 3.3 ± 0.4 mm/year^44^ and SLR has been shown to accelerate at a rate of 0.084 ± 0.025 mm/year in the 25 years leading up to 2017^21^. Based on the IPCC’s projected lower and upper-end scenarios, global SLR is expected to increase at a rate of 4–9 mm/year (RCP2.6) and 10–20 mm/year (RCP8.5), by the year 2100^17^. However, the potential impacts of SLR on intertidal ecosystems may be minimal if the rate of vertical accretion exceeds or maintains pace with the projected rates of SLR. There is currently very limited information on the maximum SLR rate at which intertidal ecosystems can adjust to SLR via accretion, without being permanently submerged.

Sediment accretion in intertidal systems is mostly associated with sediment supply, tidal inundation and frequency, plant productivity and porewater salinity^45^. Sediment accretion rates for intertidal saltmarsh ecosystems are reported to range from 0.3 to 0.8 mm/year for Europe, USA and Australia^46^, while some studies have reported up to 1.3 mm/year for USA^47^. Accretion rates are highly variable in different geomorphic settings and large discrepancies exist in the literature. For example, studies have shown that saltmarsh accretion rates have not been sufficient to keep pace with SLR over the last century and accretion rates may not be able to keep pace with future SLR even under the most optimistic IPCC SLR scenario^48^. A recent study suggests that mangroves may not be able to sustain sufficient accretion when relative SLR exceeds 6.1 mm/year (with current sea levels expected to exceed 7 mm/year by 2050 under high emissions)^49^. In summary, based on our understanding of current accretion rates and limited sediment supply (partly due to anthropogenic flow attenuation via upstream structures), vegetated intertidal ecosystems are unlikely to maintain accretion with future SLR (i.e. resulting in widespread submergence of wetlands)^7,50^. In these circumstances, the Tidal Replicate Method could be utilised to adaptively manage the tidal regime in line with accretion and SLR rates.

### Global implications

**Figure 3:**
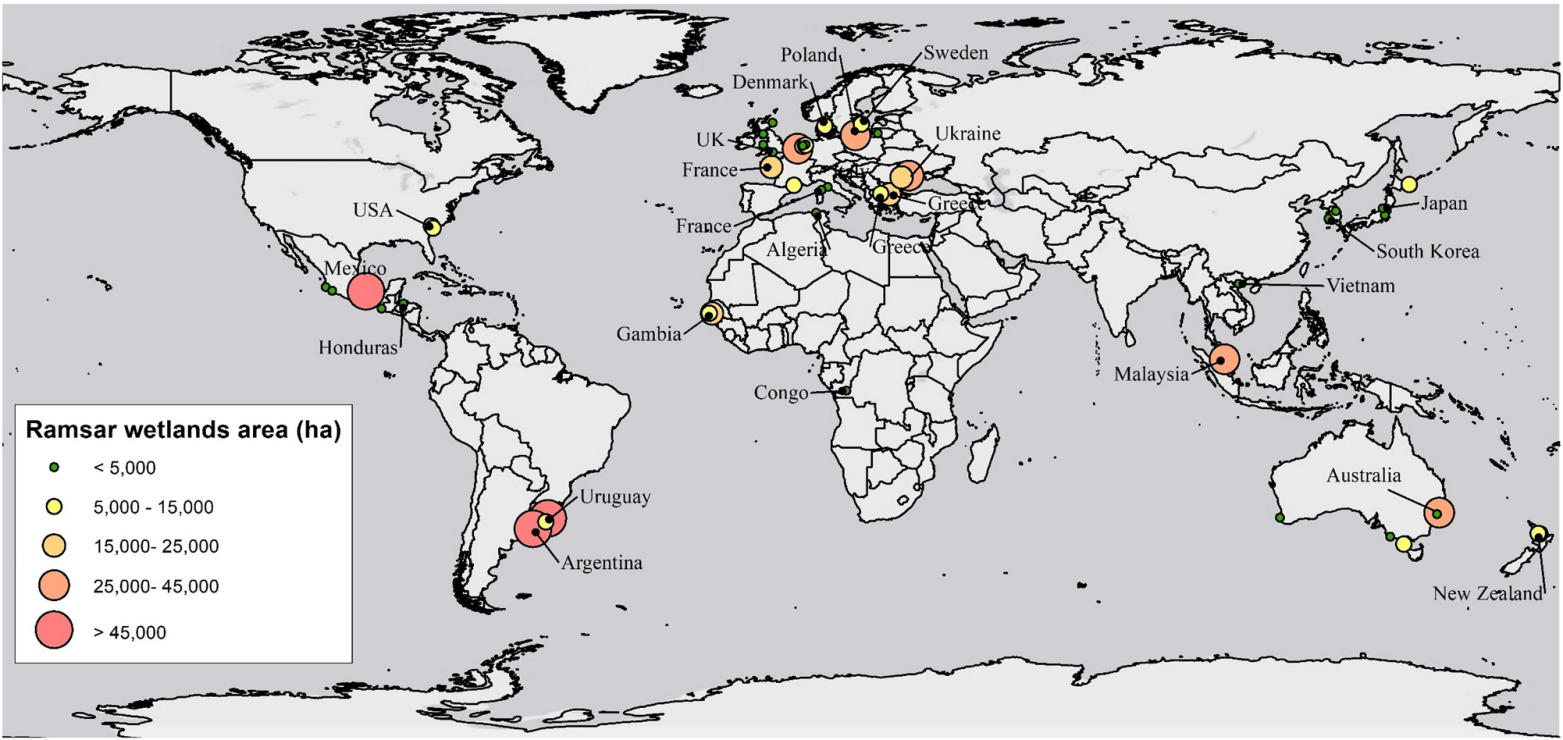
Ramsar wetlands location and relative area that can potentially be preserved against SLR through the Tidal Replicate Method. Map was created using Arc-GIS 10.5 (http://www.esri.com).

Ramsar convention listed coastal wetlands provide many valuable ecosystem services, however, their value and benefits are usually underestimated^51^. A Ramsar wetland provides ecosystem services estimated at $194,000 ha^−1^ year^−1^ (USD)^6,52^. Millions of hectares of Ramsar wetlands are currently under threat from SLR and no long-term solution has been proposed or action taken to protect these high priority wetlands from being lost. The Tidal Replicate Method, where applicable, is a feasible solution for protecting or preserving these ecosystems. Here, Ramsar listed wetlands worldwide were examined to determine if the Tidal Replicate Method is broadly transferrable to these wetlands. The Centre for International Earth Science Information Network (CIESIN, Columbia University, 2013) and Ramsar Convention data repository (https://ramsar.org/) were used to identify Ramsar wetlands worldwide. Coastal and intertidal wetlands with a minimum elevation of 3 m (approximately equal to the higher end SLR scenario), were filtered resulting in 480 Ramsar wetlands (from the initial 1800) in all continents. Thereafter, the geometry and geographical location of the short-listed sites were investigated to determine whether the Tidal Replicate Method is applicable (e.g. each Ramsar wetland site was assessed to ensure that a single channel was available to control the hydraulics). This comprehensive survey identified 32 Ramsar listed sites over six continents that can potentially utilise the Tidal Replicate Method to adapt to SLR. If an automated tidal control system (e.g. SmartGate) is implemented at these sites, over 1,184,000 ha of wetlands of international significance can be preserved from partial or full permanent inundation in response to accelerating SLR (Fig. 3). This equates to an ecosystem service savings of $230 billion USD per year versus the status quo or no action strategy (Table 2).

**Table 2:** Ramsar wetlands area (ha) which are potentially suitable for implementing the Tidal Replicate Method and their associated annual ecosystem services value.

Region	Area (ha)	Value (US $/year) × 10^9^
Africa	25,669	4.9
Asia	2861	0.5
Europe	147,931	28
Latin America and the Caribbean	662,955	129
North America	272,786	53
Oceania	72,267	14
Global	1,184,470	230

## Conclusion

SLR is threatening high priority vegetated intertidal ecosystems and unless widespread action is taken, thousands of hectares of wetland ecosystems may be lost. Currently, there is no global strategy in place to conserve or adaptively manage high value vegetated intertidal ecosystems. As these threats are focused on the hydrologic regime, a reasonable solution is to actively manage a site’s hydrology to ensure it can adaptively replicate the desired onsite conditions. Here, we present an eco-engineering solution, the Tidal Replicate Method, that can protect vegetated intertidal ecosystems by mimicking natural tidal conditions. The method is based on the inundation depth and frequency requirements of the desired vegetation type and establishes a synthetic tidal regime, implemented via an automated tidal control system (SmartGate). This novel method was implemented at a test site and demonstrated positive results. The method allows the site to be adaptively managed as sea levels or net accretion rates change with time. Worldwide, we estimate over 1,184,000 ha of high priority coastal wetlands can be preserved if the Tidal Replicate Method is adopted in other locations with similar settings.

## Materials and methods

### Study site

An intertidal temperate coastal wetland located at Kooragang Island (Hunter Wetlands National Park; − 32.866707S, 151.715561E), approximately 7 km upstream of the oceanic entrance of the Hunter River estuary, Newcastle, Australia, was chosen as the study site to implement the method. The Hunter River estuary is a wave-dominated barrier estuary with a trained and continuously dredged entrance, subject to a semi-diurnal tidal regime with a maximum amplitude of approximately 2 m^53^. The site is recognised as a Ramsar site of international importance. The location and characteristics of the site ensure it can be used as an example to replicate SLR impacts. The site’s (wetland) catchment is 24 hectares, low-lying (median elevation is 1.2 m) and has no upstream freshwater inputs. The wetland has a single estuarine channel (known as Fish Fry Creek) that is 170 m long, 10 m wide and 1.0 m deep at low tide level^7,54^, and connects to the south arm of the Hunter River estuary. The channel connects the estuary to the intertidal wetland which covers an area of 112,450 m^2^. The site experiences a temperate climate and on average receives 1122 mm rainfall annually. Temperatures at the site range from 18 to 27 °C in summer (December—February) and 7 to 17 °C in winter (June – August) (Bureau of Meteorology; http://www.bom.gov.au).

In the twentieth century, levees and internal drainage were implemented in this region to create a flood detention system which resulted in tidal waters being excluded from the wetland^55^. Following coastal wetland rehabilitation works at the area in the early 2000s, tidal flow was reintroduced to the site. However, changes in the site’s hydrology and topography favoured the expansion of mangroves, resulting in extensive loss of saltmarsh vegetation^56^. This change also affected the wetland ecosystem function including species habitats (decline in migratory shorebirds and frogs)^57^. In all, these actions resulted in a site that under natural conditions (e.g. the existing tidal regime) encouraged non-saltmarsh vegetation expansion and was not suitable for saltmarsh growth despite it historically being an important saltmarsh location for migratory shorebirds^40,58^. As such, the site was experiencing deeper tidal inundation patterns than desired, similar to that experienced with SLR, hence, making it an ideal location to trial the Tidal Replicate Method.

### Vegetation elevation and tidal planes

Field campaigns were carried out between 3rd–9th October 2016 to survey saltmarsh and mangrove tidal range and land surface elevations at the study site. In addition, other reference sites, where hydrological processes were unaffected by human activity, were also sampled across the lower Hunter River estuary. Seven nearby sites were investigated across the lower estuary, including areas on Hexham Island, Kooragang Island and Tomago Wetlands. The results from the survey were then used to determine the tidal range and topography that promotes saltmarsh vegetation growth (Supplementary Figure 4). The sediment supply rates at the study and the reference sites were known to be similar (i.e. statistically not significant)^59^. Survey points taken at each site were identified by a tagging system and grouped based on three categories; (i) saltmarsh and (ii) upper and (iii) lower bounds of mangrove stands. Over 500 points of saltmarsh and mangrove populations were surveyed at the seven sites during the field investigation. All points were surveyed to AHD using a Trimble 5800 RTK-GPS (real-time kinematic global positioning system), accurate to less than ± 20 mm. To generate near future time series tidal water elevations for the study site to develop the synthetic tidal regime, a calibrated hydrodynamic model of the Hunter River estuary developed by the Water Research Laboratory, UNSW, Sydney was utilised^60^.

### Digital elevation model and vegetation ground-truthing

A total of seven drone surveys over a 3-year period were conducted at the site to determine surface elevation through photogrammetry and vegetation development by multispectral data. Drone surveys were conducted in February and October 2017, and April and August 2018, and April and December 2019 and August 2020. For each drone survey, an eBee RTK survey grade aerial drone was flown over the site and the data was processed using the Pix4D advanced photogrammetry software to create a digital elevation model. A total of six ground control points were distributed around the site during each survey to increase the accuracy of the drone survey. Using the same software, a high resolution, geo-rectified ortho-mosaic was produced.

On-ground vegetation sampling was carried out to ground-truth drone surveys for the presence/absence of saltmarsh vegetation. There was no saltmarsh at the start of rehabilitation process. Nine field sampling events over a 3-year period in November 2017, February, June and November 2018, March, July and November 2019, and February and December 2020 were undertaken. Sampling was completed in the low, middle, and higher marsh zones based on tidal inundation depth and frequency. At each zone, 25 random 1 m^2^ quadrats were placed to measure vegetation species and cover with 75 quadrats for the entire site. Each quadrat location was marked with GPS coordinates and marker pegs for consecutive sampling events.

### Synthetic tidal regime

The synthetic tidal regime was based on local estuary data and reference sites with unimpeded tidal flushing and known flushing conditions. The number of tides and inundation levels for the study site were estimated based on the relationship between tidal planes, topography, and vegetation hydrological requirements. Based on site specific topographic conditions, the base water level (1st trigger level—the deepest water depth that stays in channel before flowing overbank) was determined. This base water level corresponded to the desired water level to be imposed at the site (e.g. the median level of saltmarsh determined based on vegetation elevation survey). The hydroperiod at the site in the synthetic tidal regime (exceedance probability), was equal to the time water levels were higher than the equivalent level in a natural tidal regime at the reference sites. The number of tides per year to reach a certain peak (trigger) were estimated as the number of times water passes a trigger over the total number of tides in a year (~ 700).

Additional trigger levels can be created to increase the control over inundation depths over time and be used to adaptively manage the tidal signal at the site (avoid the creation of non-salt marsh species that would happen naturally). Thereby, the tidal signal is artificially lowered/manipulated to generate a site-specific tidal regime within the wetland that matches the natural tidal hydroperiod observed at nearby reference sites (i.e. excludes the tidal regime that would naturally want to exist onsite). The developed synthetic tidal regime is designed such that trigger levels are as close as possible to natural tide levels. Water levels immediately before and after the SmartGate hydraulic structure were measured using Solinst Levelogger Edge Model 3001 (Solinst Canada Ltd., Georgetown, Canada) data loggers with an accuracy of ± 5 mm to ensure desired trigger levels were achieved.

## Supplementary Information

Supplementary Information.
