# Supplementary material for: Coastal wetlands can be saved from sea level rise by recreating past tidal regimes
Source: Sci Rep. 2021 Jan 13;11:1196. doi: 10.1038/s41598-021-80977-3 (PMC7807073; doi:10.1038/s41598-021-80977-3)
Supplement: Supplementary file 1 — Supplementary Information. [file 41598_2021_80977_MOESM1_ESM.docx]

Supplementary Information for:

**Coastal wetlands can be saved from sea level rise by recreating past tidal regimes**

Mahmood Sadat-Noori^1*^, Caleb Rankin^2^, Duncan Rayner^1^, Valentin Heimhuber^1^, Troy Gaston^2^, Christopher Drummond^1^, Anita Chalmers^2^, Danial Khojasteh^1^, and William Glamore^1^

*^1^Water Research Laboratory, School of Civil & Environmental Engineering, UNSW Sydney, NSW 2052, Australia*

*^2^School of Environmental and Life Sciences, University of Newcastle, Newcastle, NSW, Australia*

*Corresponding author

Mahmood Sadat-Noori, Water Research Laboratory, School of Civil & Environmental Engineering, University of New South Wales, 110 King St., Manly Vale, NSW, 2093, Australia

Email: m.sadat-noori@unsw.edu.au

Tel: (+61) 2 8071 9879

**Supplementary Figures**

Sediment, water quality and groundwater regime between broader and study site must match

Intertidal site under threat from sea level rise

(not open coastal)

Determine site topography and geometry

Assess the vegetation and salinity regime of the broader area to establish reference site conditions

Design a synthetic tidal regime for the study site

Assess the inundation regime of the study site compared to target tidal regime

Analyse the relationship between tidal dynamics and vegetation communities for all sites to establish target tidal regime

Map vegetation against topography for reference sites

Assess the tidal hydroperiod of both the reference sites and study site boundary

Create a new tidal regime for the study site

**Supplementary Figure 1.** A chart-based diagram illustrating the methodology steps of the proposed Tidal Replicate Method. Chart was created using Sigmaplot 14 ([www.systatsoftware.com](http://www.systatsoftware.com)).


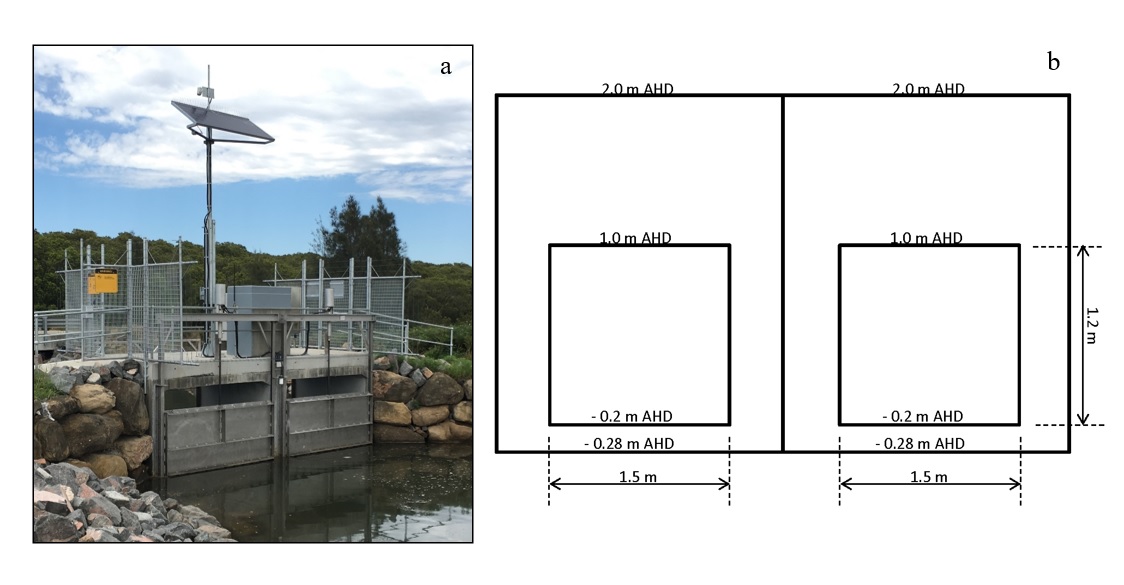


**Supplementary Figure 2.** The automated tidal control system “SmartGate” a) schematic and b) design. Photo was taken by authors and graph was created using Sigmaplot 14 ([www.systatsoftware.com](http://www.systatsoftware.com)).


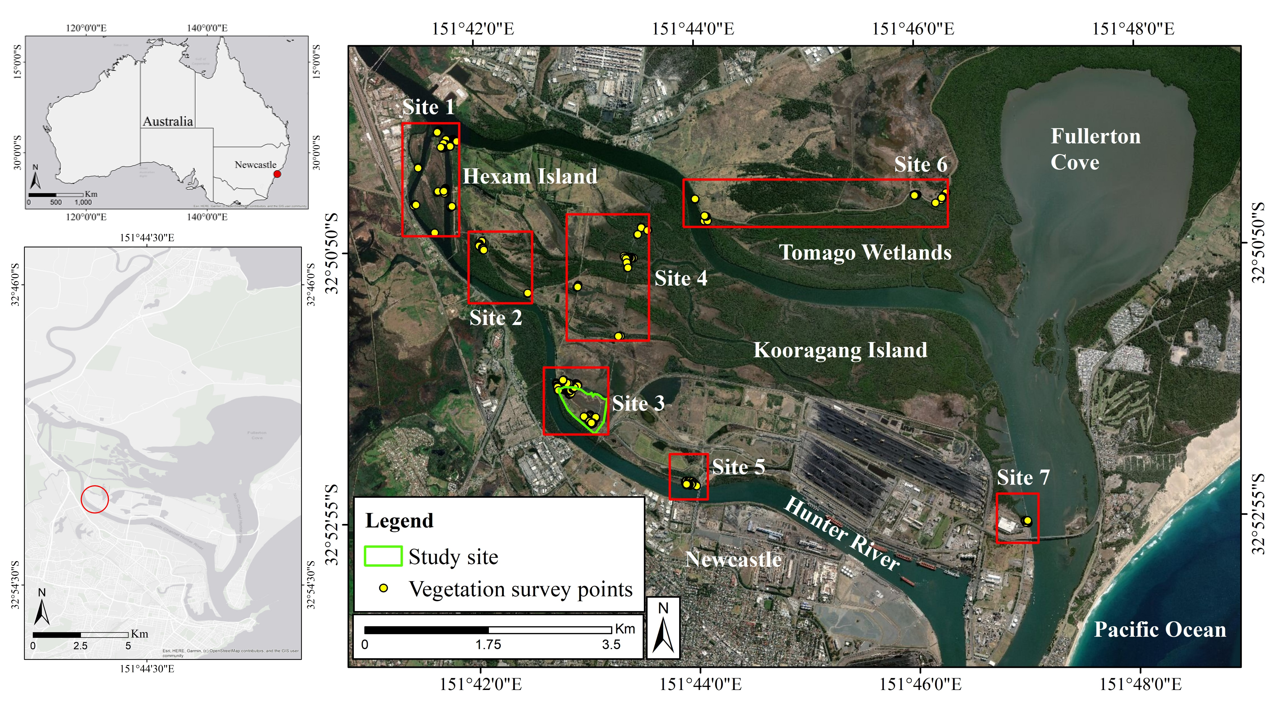


**Supplementary Figure 3.** The map of the site where the Tidal Replicate Method was applied and seven survey locations across the Hunter River Estuary, Newcastle, AUS were vegetation and elevation surveys were carried out. Map was created using Arc-GIS 10.5 (<http://www.esri.com>).


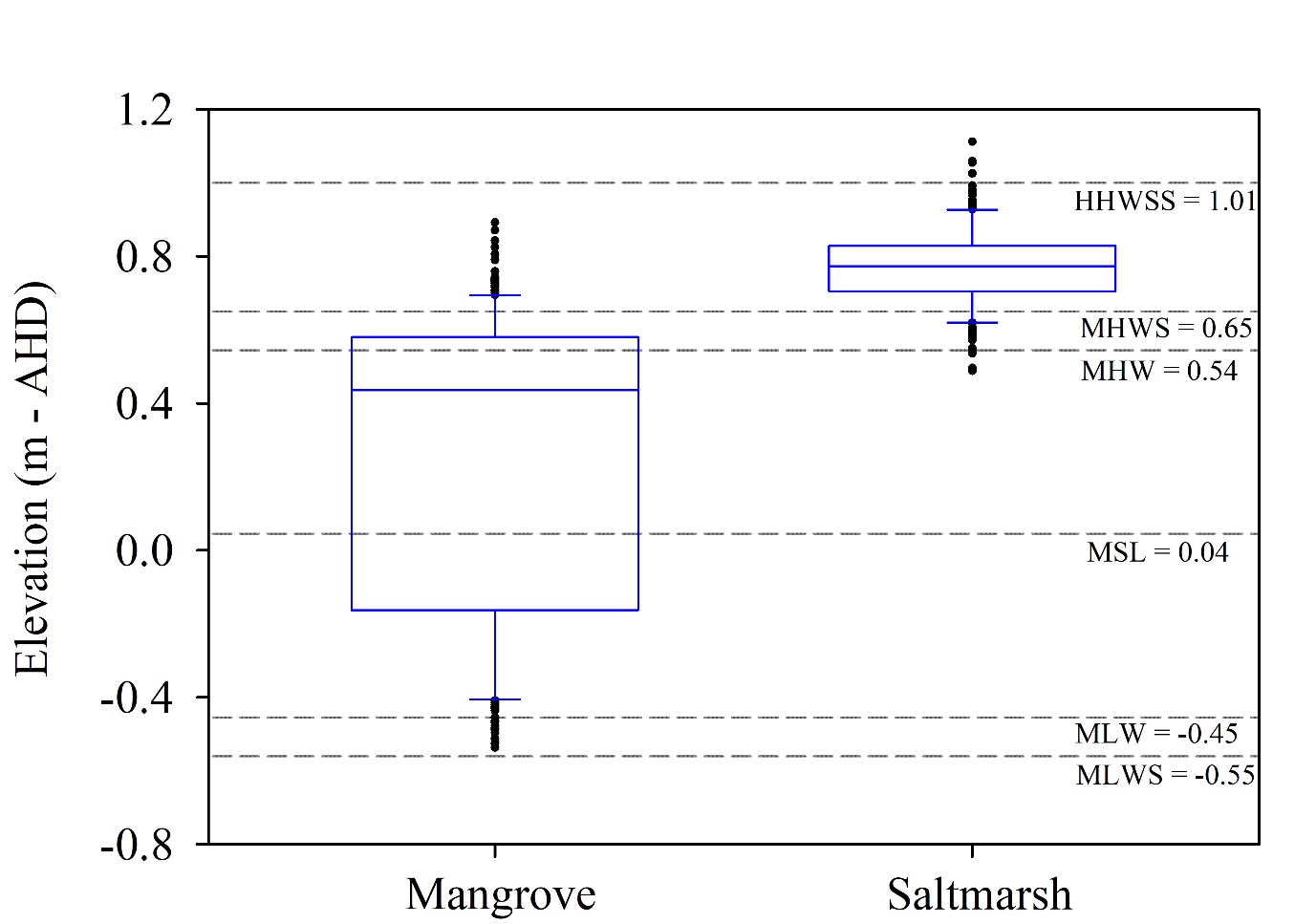


**Supplementary Figure 4.** Saltmarsh and mangrove elevations for survey points in the lower Hunter River Estuary (reference sites) and tidal plane elevation. High High-Water Solstice Springs (HHWSS), Mean High Water Springs (MHWS), Mean High Water (MHW), Mean Seal Level (MSL), Mean Low Water (MLW), Mean Low Water Springs (MLWS). Graph was created using Sigmaplot 14 ([www.systatsoftware.com](http://www.systatsoftware.com)).


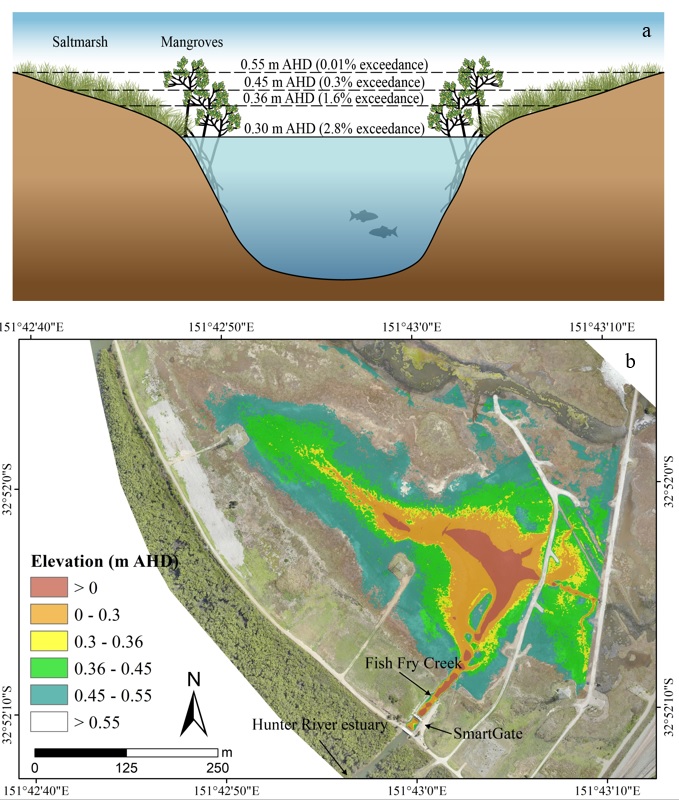


**Supplementary Figure 5.** a) Expected vegetation development areas and (b) tidal inundation extents based on elevation surveys and the synthetic tidal regime. Note that numbers applied here for this site are just applicable to this site and for creating saltmarsh. If alternative species which requires different tidal elevations to thrive were desired, number must be adjust accordingly. Figure 5a was created using Adobe Illustrator 23.0.1 (<https://www.adobe.com>) and the map was created using Arc-GIS 10.5 (<http://www.esri.com>).


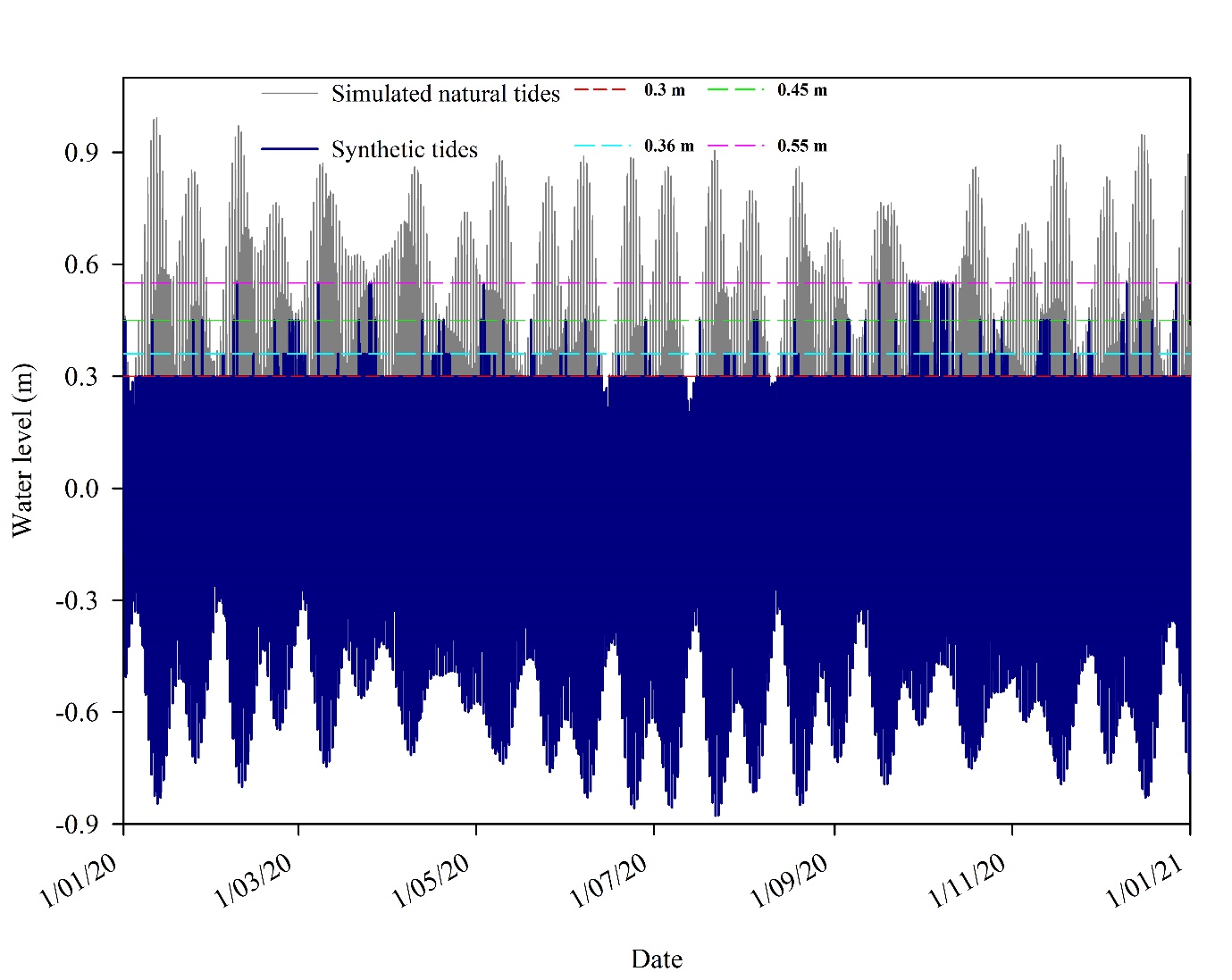


**Supplementary Figure 6.** Simulated natural and developed synthetic tidal regime for the study site. Graph was created using Sigmaplot 14 ([www.systatsoftware.com](http://www.systatsoftware.com)).

**Supplementary Table**

**Supplementary Table 1.** Tidal Planes for the study site.

| Tidal Plane | Annual Average Elevation (m AHD) |
| --- | --- |
| H.H.W.S.S. | 1.002 |
| M.H.W.S. | 0.652 |
| M.H.W. | 0.547 |
| M.S.L. | 0.048 |
| M.L.W. | -0.452 |
| M.L.W.S. | -0.558 |

Highest high-water springs; mean high water springs (M.H.W.S.); Mean high water (M.H.W.); Mean seal level (M.S.L.); Mean low water (M.L.W.); Mean low water springs (M.L.W.S.). (MHL Report 2053, 2016).

**Supplementary Table 2.** Advantages and disadvantages of different preservation methods regarding SLR.

| Strategy |  | Advantages | |  | Disadvantages | | |  | Relative costs |
| --- | --- | --- | --- | --- | --- | --- | --- | --- | --- |
|  |  | Adaptive management | Low maintenance |  | Change in soil characteristics | Fish barrier | Soil transport from other areas |  |  |
| Abandonment |  | 🗴 | 🗴 |  | 🗸 | 🗴 | 🗴 |  | No cost |
| Sediment Supply (vertical accretion) |  | 🗴 | 🗴 |  | 🗸 | 🗸 | 🗸 |  | High |
| Retreat Landward |  | 🗴 | 🗴 |  | 🗸 | 🗴 | 🗸 |  | Medium |
| Tidal Replicate Method |  | 🗸 | 🗸 |  | 🗴 | 🗴 | 🗴 |  | Low |
